# Lower Limb Doppler Ultrasound Prior to Pneumatic Compression for the Prevention of Pulmonary Embolism in Gynecological Cancer Patients: A Retrospective Cohort Study

**DOI:** 10.7759/cureus.66841

**Published:** 2024-08-14

**Authors:** Wala Mehros, Hatim Al-Jifree, Arwa Saber, Anfal Saber, Reham Makki, Batoul Fallatah, Alqassem Y Hakami, Muhammad A Khan

**Affiliations:** 1 Obstetrics and Gynaecology, King Saud Bin Abdulaziz University for Health Sciences College of Medicine, Jeddah, SAU; 2 Oncology, King Abdulaziz Medical City, Ministry of National Guard Health Affairs, Jeddah, SAU; 3 Oncology, King Abdullah International Medical Research Center, Jeddah, SAU; 4 Obstetrics and Gynaecology, King Abdulaziz Medical City, Ministry of National Guard Health Affairs, Jeddah, SAU; 5 Research Office, King Abdullah International Medical Research Center, Jeddah, SAU; 6 Research Office, King Saud Bin Abdulaziz University for Health Sciences College of Medicine, Jeddah, SAU; 7 Medical Education, King Saud Bin Abdulaziz University for Health Sciences College of Medicine, Jeddah, SAU

**Keywords:** deep-vein thrombosis, surgery, oncology gynecology, pulmonary embolism, deep vein thrombosis, leg doppler

## Abstract

Introduction: Venous thromboembolism, which includes deep-vein thrombosis and pulmonary embolism, is the third most common cardiovascular disease after myocardial infarction and stroke. This study aimed to determine the effect of Doppler ultrasound on the prophylaxis and prevention of postoperative venous thromboembolism in gynecological oncology.

Method: This is a retrospective cohort study of procedures performed at King Abdulaziz Medical City in Jeddah, Saudi Arabia, between 2016 and 2021. The study included all patients diagnosed with gynecological cancers who underwent major cancer resection. A total of 295 eligible patients were enrolled and divided into two groups: the first group consisted of 104 patients who were screened for deep vein thrombosis using lower limb Doppler ultrasound prior to their gynecologic oncology surgery, while the second group included 190 patients who were not screened.

Results: The prevalence of pulmonary embolism and/or deep vein thrombosis was found to be eight out of 104 patients (7.7%) in the group screened for deep-vein thrombosis using lower limb Doppler ultrasound prior to their gynecologic oncology surgery. In the group that was not screened, one out of 190 patients (0.5%) developed deep vein thrombosis. The prevalence of postoperative pulmonary embolism and/or deep-vein thrombosis was reported in four out of 104 screened patients (3.8%) and in three out of 190 patients (1.6%) in the unscreened group.

Conclusion: This study concluded that Doppler screening did not change the incidence of pulmonary embolism and/or deep-vein thrombosis postoperatively, but it may be helpful in detecting these conditions preoperatively. Therefore, Doppler screening for deep-vein thrombosis before surgical procedures in gynecological oncology could be considered after clinical assessment of the patient. To improve the study and address its limitations, a larger sample size would help to further investigate and identify relevant factors and determine their significance.

## Introduction

Venous thromboembolism (VTE) consists of two related conditions: deep vein thrombosis (DVT) and pulmonary embolism (PE) [1]. VTE is the third most common cardiovascular disease after myocardial infarction and stroke [2]. The overall incidence of VTE in the general population of the United States ranges from 0.1% to 0.2% [3], and approximately 25,000 people are affected annually in Saudi Arabia [4]. Moreover, the incidence of VTE is significantly higher in cancer patients compared to the general population [5]. VTE is the second leading cause of death in cancer patients, following cancer metastasis [6]. The risk factors for developing VTE are particularly prevalent in patients with ovarian, brain, lung, and pancreatic cancers [5]. Among gynecological cancers, the highest incidence of VTE is observed in patients with ovarian cancer, followed by those with uterine cancer [7]. Additionally, patients who undergo minimally invasive surgery for gynecological cancers have a lower incidence of VTE than those who undergo open surgery [7].

Although VTE has been associated with significant morbidity and mortality following gynecological surgery, its incidence has decreased with the use of prophylactic measures such as sequential compression devices (SCD), unfractionated heparin (UFH), and low-molecular-weight heparin (LMWH) [8]. The use of SCD is thought to prevent DVT early during surgery, reducing the risk of progression to PE. VTE is initially diagnosed through patient history and physical examination, followed by Doppler ultrasound (US) for suspected DVT, with computed tomography (CT) employed for suspected PE cases [9].

A prospective study conducted in Kansas, United States, reported that Doppler US is effective in the early detection and management of postoperative DVT [10]. The study involved performing Doppler screening before cosmetic surgery, the day after surgery, and one week postoperatively, with affected patients receiving weekly scans. Of the 1000 patients screened, nine (0.9%) had positive DVTs: two the day after surgery and six one week postoperatively. This indicates the efficacy of Doppler US in diagnosing postoperative DVT [10].

Another study assessed the prognostic significance of postoperative PE after gynecologic oncology surgery, finding that among 134 patients who experienced lower limb symptoms, 38 were found to have DVT following Doppler US screening [11]. This suggests that patients undergoing gynecologic oncology surgery are at risk of developing DVT, which in turn increases their susceptibility to PE, particularly with the use of SCDs. The periodic compressions generated by SCDs in the lower limbs may dislodge an embolus in the presence of an existing DVT, leading to PE. These findings were supported by a subsequent two-year study that compared PE incidence in patients with benign versus malignant conditions who underwent gynecologic oncology surgery and received VTE prophylaxis with intermittent pneumatic compression [12]. The results showed a higher incidence of PE in cancer patients compared to those with benign conditions (4.1% vs. 0.3%, respectively) and identified cancer and age (above 60) as significant risk factors [13].

Furthermore, a recent study explored the frequency of preoperative asymptomatic VTE in gynecological cancer patients and its link to postoperative PE. It identified VTE in 7.3% of cervical cancer cases, 11.5% of endometrial cancer patients, and 27% of ovarian cancer patients [14]. This highlights the importance of considering VTE screening before surgery in gynecologic oncology patients to avoid complications from asymptomatic preoperative VTE.

Thus, the exact effect of Doppler US in screening for DVT before a surgical procedure in gynecology oncology patients in terms of the risk of PE is not clear. Therefore, the aim of this study was to compare the outcomes of VTE in patients with gynecological cancers who underwent lower limb Doppler US prior to the use of intra-operative pneumatic compression with patients who did not in order to determine whether the use of Doppler US decreases the risk of PE. The results will help to clarify the need for pre-operative Doppler US for gynecology oncology patients and whether it should be integrated into surgical protocols and pre-operative assessment.

## Materials and methods

This study was conducted in the Gynecology Oncology Department at King Abdulaziz Medical City in Jeddah (KAMC-J), Saudi Arabia, and was approved by the Institutional Review Board.

The inclusion criteria for this study encompassed all patients diagnosed with gynecological cancers who underwent major cancer resection at KAMC-J between 2016 and 2021, as well as patients diagnosed with DVT within six weeks prior to surgery who received treatment. The exclusion criteria included patients with active or newly diagnosed DVT, those undergoing active anti-thrombolytic treatment, day surgery patients, and those with hematological diseases. This study recruited all subjects who met the inclusion criteria, so no sampling technique was required.

The sample size was calculated to be 300 using Rasoft software (Raosoft, Inc., Seattle, Washington), with a margin of error of 1.70% and a 95% confidence level, based on a population of 700 patients and a PE incidence of 4.1% [12].

Data were obtained from the institution’s electronic healthcare system. Generalization bias was minimized in this study. The variables collected included demographic data (age, gender, weight, height, and body mass index (BMI)). Postoperative DVT and PE were diagnosed within 4-8 weeks, with DVT diagnosed using Doppler ultrasound and PE diagnosed using computed tomography (CT) angiography. Microsoft Excel (Microsoft Corporation, Redmond, Washington) was used for data entry, and IBM SPSS Statistics for Windows, Version 25 (Released 2017; IBM Corp., Armonk, New York) was used for analysis. Qualitative and demographic data are presented as frequencies and percentages, while quantitative data are presented in tables. The independent t-test and Mann-Whitney test were used for continuous variables, and the Fisher exact test was used for categorical variables to determine associated factors. A p-value <0.05 was considered statistically significant.

## Results

A total of 295 patients diagnosed with gynecological cancers and treated surgically were included in this study. The study consisted of two groups: Arm A included 104 patients who were screened for DVT via Doppler ultrasound, and Arm B included 191 patients who were not screened for DVT prior to intraoperative pneumatic compression. The demographic data for these patients are detailed in Table 1.

**Table 1:** Participant demographic data IQR: interquartile range.

	Median	IQR	Min	Max
Age	62	52-62	16	90
Weight	77	63.4-77	32	153.5
Height	154	150-154	135	180
BMI	32.4	26.2-38.4	19	66.4

**Figure 1:**
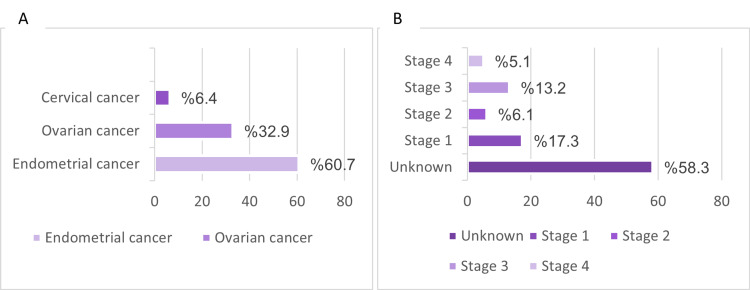
Types and stages of gynecological cancers in patients undergoing major cancer resection surgery

The types of gynecological cancer among the studied patients varied, with endometrial cancer accounting for 60%, ovarian cancer for 32.9%, and cervical cancer for 6.4%. The stages of cancer were classified into stages 1-4, with an additional category for unknown stages. The majority of patients (58.3%) were classified as having an unknown stage, which could be attributed to the fact that most cancer stages are determined after surgery (Table 2, Figure 1).

**Table 2:** Prevalences of reported risk factors in the sample of patients with gynecological cancers undergoing major resection surgery, including medical history (A), cancer type and staging (B), and drug history (C) CVD: cardiovascular disease, DVT/PE: deep vein thrombosis/pulmonary embolism, HRT: hormone replacement therapy.

Variable	n	%
(A) Medical history		
Diabetes (n=294)		
Yes	129	43.9
No	165	56.1
Hypertension (n=294)		
Yes	138	46.9
No	156	53.1
Dyslipidemia (n=294)		
Yes	72	24.5
No	222	75.5
CVD (n=294)		
Yes	24	8.2
No	270	91.8
Vascular disease (DVT/PE) (n=294)		
Yes	31	10.5
No	263	89.5
Hematological disorder (n=294)		
Yes	10	3.4
No	284	96.6
Smoker (n=295)		
Yes	2	0.7
No	293	99.3
(B) Cancer type and staging		
Cancer (n=295)		
Yes	236	80
No	59	20
Type of cancer (n=295)		
Endometrial cancer	179	60.7
Ovarian cancer	97	32.9
Cervical cancer	19	6.4
Stage of cancer (n=295)		
Unknown	172	58.3
Stage 1	51	17.3
Stage 2	18	6.1
Stage 3	39	13.2
Stage 4	15	5.1
(C) Drug history		
Blood thinners (n=293)		
Yes	32	10.9
No	261	89.1
HRT (n=294)		
Yes	7	2.4
No	287	97.6
Glucocorticoids (n=294)		
Yes	17	5.8
No	277	94.2

**Figure 2:**
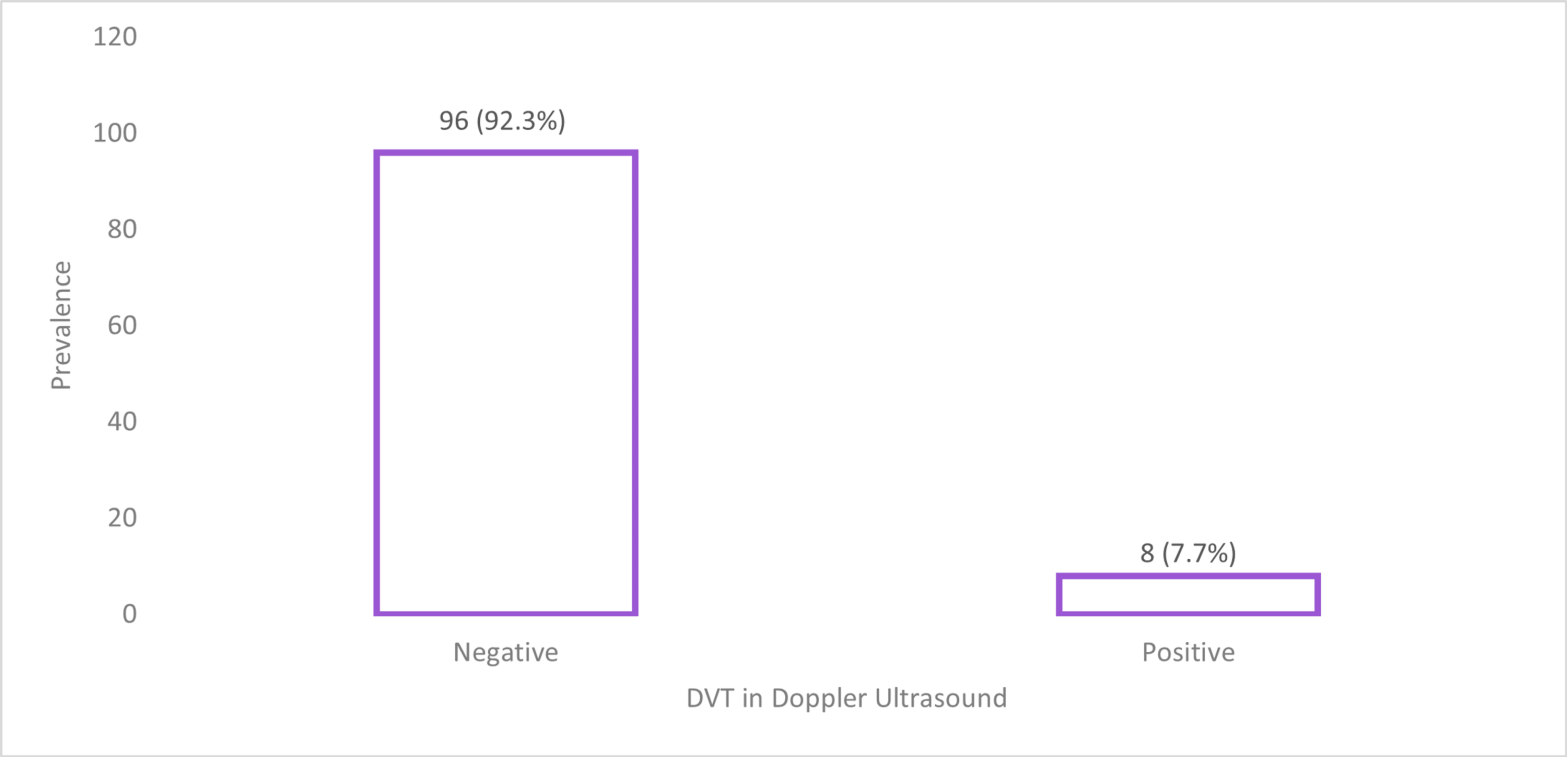
Leg Doppler US results prior to gynecological oncology surgeries and deep vein thrombosis US: ultrasound, DVT: deep vein thrombosis.

In our study sample, we accounted for any medication consumed by patients prior to surgery. It was found that seven patients (2.4%) were undergoing hormone replacement therapy (HRT), 17 patients (5.8%) were taking glucocorticoids, and 32 patients (10.9%) were taking blood thinners (Table 2C). In Arm A, of all patients who were screened for preoperative DVT, nine (8.6%) reported positive DVT in the leg Doppler US, while 96 (92.3%) were negative for DVT (Table 3, Figure 2).

**Table 3:** Leg Doppler US prior to gynecological oncology surgeries and deep vein thrombosis US: ultrasound, DVT: deep vein thrombosis.

Leg Doppler	DVT	n	%
Screened by leg Doppler prior to surgery	Yes	8	7.7
	No	96	92.3
	Total	104	100
Not screened by leg Doppler prior to surgery	Yes	1	0.5
	No	189	99.5
	Total	190	100

In Arm A, four patients (3.8%) developed postoperative DVT/PE, while 92 patients (88.5%) reported no postoperative complications. In contrast, Arm B had three patients (1.6%) who developed DVT/PE, with 178 patients (93.2%) reporting no postoperative complications (Table 4).

**Table 4:** Leg Doppler US prior to gynecological oncology surgeries and post-operative complications US: ultrasound, DVT: deep vein thrombosis, PE: pulmonary embolism.

Leg Doppler	Postoperative DVT and PE	n	%
Screened by leg Doppler prior to surgery	No	92	88.5
	DVT and PE	4	3.8
	Others	8	7.7
	Total	104	100
Not screened by leg Doppler prior to surgery	No	178	93.2
	DVT and PE	3	1.6
	Others	10	5.2
	Total	191	100

This study identified potential risk factors for DVT in patients undergoing gynecologic oncology surgery. We found a high prevalence of comorbidities: 46.9% of patients had hypertension (HTN), 43.9% had diabetes mellitus (DM), and 24.5% had dyslipidemia (DLP) (Table 2). Among the patients with pre-operative DVT, six had HTN, four had DM, three had DLP, and three had cardiovascular disease (CVD) (Table 5). These findings suggest a possible correlation between these comorbid conditions and the risk of DVT in this patient population.

**Table 5:** Risk factors and preoperative deep vein thrombosis DVT: deep vein thrombosis, CVD: cardiovascular disease, DVT/PE: deep vein thrombosis/pulmonary embolism.

Risk Factor	DVT			
	Yes (N= 9), n (%)	No (N = 284), n (%)	Total (N = 293)	p
Diabetes				>0.99
Yes	4 (3.1)	124 (96.9)	128	
No	5 (3.0)	160 (97.0)	165	
Hypertension				0.313
Yes	6 (4.4)	131 (95.6)	137	
No	3 (1.9)	153 (98.1)	156	
Dyslipidemia				0.694
Yes	3 (4.2)	69 (95.8)	72	
No	6 (2.7)	215 (97.3)	221	
CVD				0.029
Yes	3 (12.5)	21 (87.5)	24	
No	6 (2.2)	263 (97.8)	269	
Vascular disease (DVT/PE)				0.244
Yes	2 (6.5)	29 (93.5)	31	
No	7 (2.7)	255 (97.3)	262	
Hematological disorder				0.033
Yes	2 (20)	8 (80)	10	
No	7 (2.5)	276 (97.5)	283	
Smoker				>0.99
Yes	0 (0)	2 (100)	2	
No	9 (3.1)	283 (96.9)	292	

The results of this study suggest that the type of gynecological cancer is linked to the incidence of DVT. Among the patients who were screened, a higher incidence of DVT was associated with endometrial cancer, with seven patients accounting for 3.9% of cases, while the remaining cases were attributed to ovarian cancer (Table 6). Although the stage of cancer did not yield a statistically significant value, the results in the screened arm (Arm A) showed that five patients had an unknown stage of cancer, one patient was diagnosed with stage 1, two patients with stage 2, and one patient with stage 4 (Table 6).

**Table 6:** Cancer type and stage associated with deep vein thrombosis DVT: deep vein thrombosis.

Risk Factor	DVT			
	Yes (%)	No (%)	Total	p
Type of cancer				0.73
Endometrial cancer	7 (3.9)	172 (96.1)	179	
Ovarian cancer	2 (2.1)	94 (97.9)	96	
Cervical cancer	0 (0)	19 (100)	19	
Stage of cancer				0.121
Unknown	5 (2.9)	167 (97.1)	172	
Stage 1	1 (2)	50 (98)	51	
Stage 2	1 (5.6)	17 (94.4)	18	
Stage 3	0 (0)	38 (100)	38	
Stage 4	2 (13.3)	13 (86.7)	15	

This study demonstrated that Doppler screening had no impact on the postoperative occurrence of PE or DVT. However, it may be beneficial as a preoperative screening method for lower limb DVT in patients who will use SCDs during surgery. This approach could help prevent postoperative PE complications in cases where asymptomatic DVT is already present.

## Discussion

VTEs are considered life-threatening and can significantly impact morbidity and mortality, particularly in patients undergoing gynecological oncological surgeries [1]. As a prophylaxis, patients can be administered pharmacological anticoagulants as well as mechanical prophylaxis, such as SCDs [8]. However, in cases of asymptomatic pre-operative DVT, SCDs can dislodge an existing embolus. Thus, detection of DVTs by leg Doppler US, specifically prior to the use of SCDs intraoperatively, is essential to prevent possible subsequent PE complications.

Therefore, this study evaluated the efficacy of lower limb Doppler US prior to pneumatic compression in patients with gynecological cancers who undergo surgery, as well as its role in the prevention of VTEs. In addition, this study established the prevalence of DVT in patients with gynecological cancers prior to surgery diagnosed via lower limb Doppler US. Finally, it assessed the risk and prevalence of DVT/PE in patients diagnosed with gynecological cancer who undergo lower limb Doppler US prior to pneumatic compression compared with those who do not.

This study demonstrated that DVT screening in gynae-oncology pre-surgical assessment should be considered before the use of SCDs. In this study, 104 of 295 patients were screened for DVT using lower limb Doppler US prior to gynae-oncological surgery where SCDs are utilized as PE prophylaxis. The results showed that of those 104 patients, eight were found to have DVT, accounting for 7.7% (Table 3, Figure 2). This highlights the risk of patients developing PE, as the use of Doppler US to identify cases of existing DVT is not currently included in surgical protocols and guidelines. This means that some patients are exposed to SCDs without first being cleared of DVTs, making them susceptible to critical complications of PE, which can increase mortality and morbidity. Furthermore, the use of Doppler US as a screening tool allows for the administration of a therapeutic dose of heparin, rather than a prophylactic dose, to resolve the DVT and prevent possible PE complications, permitting patients to undergo surgery safely.

In addition, several factors and conditions are known to increase the risk of DVT. This study established that certain conditions may be associated with a higher risk of DVT in patients scheduled for gynecologic oncology surgeries. Co-morbid disorders, including DM, hypertension (HTN), and dyslipidemia (DLP), were highly prevalent, affecting 43.9%, 46.9%, and 24.5% of patients, respectively (Table 2). Among patients diagnosed with pre-operative DVT, six had a history of HTN, four of DM, three of DLP, and three of cardiovascular disease (CVD) (Table 5). This supports previous evidence that chronic conditions may be linked with a higher risk of VTEs. Factors that were found to have a statistically significant association with VTEs are CVD (p = 0.029) and hematological disorders (p = 0.033) (Table 5). This suggests that patients with a history of CVDs and hematological disorders are at a higher risk of developing DVT. Thus, Doppler US screening for DVTs may benefit these high-risk groups. The use of risk assessment tools, such as those recommended by the American College of Chest Physicians (AACP), i.e., the Caprini score or Rogers score, which take into account patients’ demographic data as well as their medical history [14,15], can aid in identifying high-risk patients who should be considered for DVT screening prior to surgery. The results of this study, in combination with those of related studies, may be used to generate a risk assessment tool specifically for gynecology oncology patients undergoing surgery; specifically, to classify patients who should be screened for DVT before surgery, considering factors such as the type and staging of cancer.

Furthermore, while it has been established that gynecological cancer is associated with VTEs, the type of cancer can also play a role. The results of this study support those of other studies showing that gynecological cancers are linked to a higher incidence of DVT. For example, a study conducted in 2009 demonstrated that cancers of the female reproductive system are significantly correlated with DVT [15]. Our statistical analysis revealed that the most frequent type of cancer in the study population was endometrial (60.7%), followed by ovarian (32.9%), and lastly cervical (6.4%) (Table 2). In addition, in patients who were screened, a higher incidence of DVT was linked with endometrial cancer, with seven patients accounting for 3.9%, and the remaining cases attributed to ovarian cancer (Table 6). In contrast, other similar studies aimed at identifying the risk factors and occurrence of pre-operative asymptomatic VTE to prevent post-operative PE complications, such as a recent Japanese study, found that DVT is highly associated with ovarian cancer, followed by endometrial and then cervical cancer [13]. This difference may be attributed to the larger sample size or perhaps a variation in regional demographics.

Moreover, the risk of VTEs may be affected by the stage of cancer at the time of operation. Although the stage of cancer did not yield a significant p-value, the results showed that the most common staging in gynecological oncology patients is ‘unknown’ (58.3%), followed by stage 1 (17.3%) (Table 2). Specifically, in patients in the screened arm (A), five patients had an unknown stage of cancer, one patient was diagnosed with stage 1, two patients with stage 2, and one with stage 4 (Table 6). Hence, patients with unidentified staging had the highest incidence of DVT prior to surgery. In comparison, existing literature indicates that factors associated with higher mortality in relation to DVT include early-stage cancer [7]. This could be due to the fact that surgical intervention in gynecological oncology is considered for the management of patients with unidentified and early staging, as it has a better prognosis, whereas surgery in later stages may have poor outcomes [12].

However, this study has certain limitations that can be attributed to several factors. For example, while this study had an appropriate sample size, it was too small for a Fisher’s exact test statistical analysis to be conducted. A future study could include a larger sample by incorporating patients from different centers or regions. Furthermore, there were a few limitations such as missing data that may have affected the results. Additionally, there was some variation with previous studies, for instance, in the data for the type and staging of gynecological cancers linked to a higher risk of DVT. Again, a larger sample size may benefit further investigation and help identify other factors involved, such as age, concurrent chronic diseases, and type of cancer. This may help to clearly highlight high-risk patients and identify those who should be screened for DVT via Doppler US.

## Conclusions

In conclusion, Doppler US screening for DVT before a surgical procedure in gynecological oncology patients can reduce the risk of PE in high-risk patients. This study showed that of the 104 patients who were screened, 7.7% were found to have DVT. The study also identified risk factors, including CVD and blood disorders. Therefore, Doppler US screening for DVT should be integrated into surgical protocols and pre-operative assessment for high-risk patients. Specifically, Doppler US has the potential to be a valuable tool in pre-operative screening for lower limb DVT in patients where the intra-operative use of SCDs is implemented, for the prevention of post-operative PE complications in cases of existing asymptomatic DVT.
